# [Retracted] Downregulation of let‑7b promotes COL1A1 and COL1A2 expression in dermis and skin fibroblasts during heat wound repair

**DOI:** 10.3892/mmr.2023.13144

**Published:** 2023-12-11

**Authors:** Jinyan Liu, Chengqun Luo, Zhaoqi Yin, Ping Li, Shaohua Wang, Jia Chen, Quanyong He, Jianda Zhou

Mol Med Rep 13: 2683–2688, 2016; DOI: 10.3892/mmr.2016.4877

Following the publication of this paper, it was drawn to the Editor's attention by a concerned reader that the western blotting data shown in Figs. 4B and 5 and the H&E immunostaining data shown in Fig 1A were strikingly similar to data appearing in different form in other articles written by different authors at different research institutes that had either already been published, or were submitted for publication at around the same time.

Owing to the fact that the contentious data in the above article had already been published prior to its submission to *Molecular Medicine Reports*, the Editor has decided that this paper should be retracted from the Journal. The authors were asked for an explanation to account for these concerns, but the Editorial Office did not receive a reply. The Editor apologizes to the readership for any inconvenience caused.

